# Transcriptome analysis of haploid male gametophyte development in *Arabidopsis*

**DOI:** 10.1186/gb-2004-5-11-r85

**Published:** 2004-10-27

**Authors:** David Honys, David Twell

**Affiliations:** 1 Institute of Experimental Botany AS CR, Rozvojová 135, CZ-165 02, Praha 6, Czech Republic; 2 Department of Plant Physiology, Faculty of Sciences, Charles University, Viničná 5, CZ-128 44, Praha 2, Czech Republic; 3 Department of Biology, University of Leicester, University Road, Leicester LE1 7RH, UK

## Abstract

**Background:**

The haploid male gametophyte generation of flowering plants consists of two- or three-celled pollen grains. This functional specialization is thought to be a key factor in the evolutionary success of flowering plants. Moreover, pollen ontogeny is also an attractive model in which to dissect cellular networks that control cell growth, asymmetric cell division and cellular differentiation. Our objective, and an essential step towards the detailed understanding of these processes, was to comprehensively define the male haploid transcriptome throughout development.

**Results:**

We have developed staged spore isolation procedures for *Arabidopsis *and used Affymetrix ATH1 genome arrays to identify a total of 13,977 male gametophyte-expressed mRNAs, 9.7% of which were male-gametophyte-specific. The transition from bicellular to tricellular pollen was accompanied by a decline in the number of diverse mRNA species and an increase in the proportion of male gametophyte-specific transcripts. Expression profiles of regulatory proteins and distinct clusters of coexpressed genes were identified that could correspond to components of gametophytic regulatory networks. Moreover, integration of transcriptome and experimental data revealed the early synthesis of translation factors and their requirement to support pollen tube growth.

**Conclusions:**

The progression from proliferating microspores to terminally differentiated pollen is characterized by large-scale repression of early program genes and the activation of a unique late gene-expression program in maturing pollen. These data provide a quantum increase in knowledge concerning gametophytic transcription and lay the foundations for new genomic-led studies of the regulatory networks and cellular functions that operate to specify male gametophyte development.

## Abstract 2

A transcriptome analysis of male gametophyte development in *Arabidopsis* uncovers distinct temporal classes of gene expression and opens the door to detailed studies of the regulatory pathways involved.

## Background

Development of eukaryotic cells towards particular cell fates is regulated by complex and dynamic changes in gene expression. These changes, when monitored on a genome-wide scale, provide a detailed framework for the analysis and modeling of cellular development. To monitor patterns of gene expression it is important to be able to isolate cells at precise stages along a developmental pathway. Well-developed procedures for cell culture and single-cell PCR techniques have allowed genome-wide changes in gene expression to be monitored during animal cell differentiation [1-4].

Transcriptomic studies of single cell types in plants have focused on diploid sporophytic cell types, including undifferentiated cell suspensions [5,6], leaf epidermal and mesophyll cells [7], stomatal guard cells [8] and cultured mesophyll cells [9,10]. These studies have provided valuable information about gene expression in single cell types; however, their coverage of the transcriptome has been limited and/or hampered by low RNA yields from individual cells, requiring mRNA preamplification steps that can bias the complementary RNA (cRNA) [11,12]. Moreover, such studies have not involved the use of the most comprehensive tools for monitoring gene expression that are now available for *Arabidopsis *- which include the Affymetrix ATH1 gene arrays. Recently, a significant advance in transcriptome analysis of plant cell types has been achieved through fluorescence-activated cell sorting of cell-type marked and protoplasted root cells using Affymetrix ATH1 micorarrays [13]. This has provided a near-comprehensive transcriptomic view of cell-fate determination at three developmental stages in five different domains of the root apex.

In contrast to such enabling technologies and procedures developed for sporophytic cell types there have been no studies that provide a genome-wide perspective of cell fate determination and differentiation during haploid gametophyte development. The haploid male gametophyte generation of flowering plants has a simple and well-defined pathway of development and consists of two- or three-celled pollen grains that deliver two sperm cells via the pollen tube to the embryo sac at fertilization. The highly reduced cell lineage and functional specialization of the male gametophyte are thought to be key factors in the reproductive fitness and evolutionary success of flowering plants. Moreover, pollen ontogeny provides an attractive model of cellular development in which to dissect the regulation of cell growth and division, cellular differentiation and intercellular communication (for reviews see [14-17]).

Recent progress in understanding of molecular and cellular aspects of pollen development has emerged from genetic studies that have identified mutants in *Arabidopsis *that affect all phases of pollen development [18-29]. In parallel, cDNA libraries and databanks have been obtained for sperm cells in maize, lily, tobacco and *Plumbago zelanica *[30-33]. Despite such advances there is limited information about developmental changes in gene expression associated with particular phases of male gametophyte development. Our objective was to develop procedures to enable the isolation of populations of microspores and developing pollen grains at precise developmental stages in *Arabidopsis *and to analyze changes in gene expression from unicellular microspores to mature differentiated pollen grains. A particular advantage of the male gametophyte generation is that developing microspores and pollen grains are symplastically isolated. This facilitates access to viable cell populations at different stages of haploid development without contaminating sporophytic cells.

Some initial progress has been made towards the definition of the male gametophytic transcriptome of *Arabidopsis *using serial analysis of gene expression (SAGE) [34] and Affymetrix AG microarrays that harbor probes for approximately 8,000 different genes [35,36]. These studies have provided valuable insight into the complexity of gene expression in mature pollen and the extent of overlap between male gametophytic and sporophytic gene expression (reviewed in [37]). However, these studies monitored the expression of only 30% of the annotated genes in *Arabidopsis *and analyzed mRNA populations only in mature differentiated pollen grains. These studies do not, therefore, provide a developmental perspective of gene expression during development and differentiation of the male gametophyte.

Here we describe spore isolation procedures for *Arabidopsis *and the use of Affymetrix ATH1 Genome Arrays to analyze transcript expression profiles throughout four successive stages of male gametophyte development in *Arabidopsis*. Isolated spore populations were large enough to enable RNA extraction for direct microarray hybridization without any preceding amplification step that could lead to bias in expression signals between stages or between genes within individual stages. Progression from proliferating microspores to terminally differentiated pollen was characterized by large-scale repression and the activation of a unique collection of late-program genes during pollen maturation. Putative male gametophyte-specific genes and distinct clusters of coexpressed genes are identified, including key groups of regulatory factors including cell cycle, transcription and translation factors. Bioinformatic and experimental data are used to address the importance of transcription and translation during pollen germination and tube growth

## Results

### Isolation and characterization of developing spores

Transcriptome profiling throughout microgametogenesis in *Arabidopsis *required the introduction of a procedure for the isolation of homogeneous populations of viable spores at precisely defined stages of development. The method was based on centrifugation of isolated mixed spores in a Percoll step-gradient [38,39]. Large homogeneous spore populations at three developmental stages were collected: uninucleate microspores (UNM), bicellular pollen (BCP) and immature tricellular pollen (TCP). In addition, a homogeneous mature pollen grain (MPG) population was isolated from open flowers according to Honys and Twell [35].

**Figure 1:**
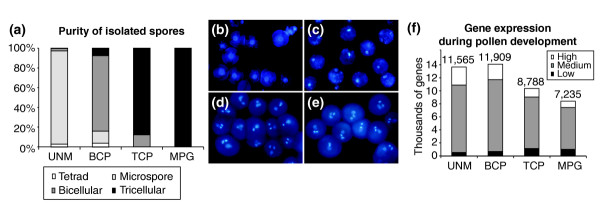
Spore isolation and numbers of genes expressed throughout *Arabidopsis *male gametophyte development. **(a) **Purity of isolated spores in each developmental stage determined by microscopy: UNM, microspores; BCP, bicellular pollen; TCP, tricellular pollen; MPG, mature pollen. **(b-e) **DAPI-stained populations of developing spores: (b) microspores; (c) bicellular pollen; (d) tricellular pollen; and (e) mature pollen. **(f) **Total number of genes expressed in developing pollen and their distribution among three relative abundance classes. Gene-abundance classes were defined as follows: high (up to 10-fold less than the maximum signal), medium (10- to 100-fold less) and low (more than 100-fold less).

Microscopic examination of isolated spore populations revealed no contaminating sporophytic cells and little or no other cellular debris (Figure 1a). Vital staining revealed more than 90% viable spores at each stage (data not shown). The purity of spore populations was evaluated by DAPI staining (Figure 1b-e). The UNM population was the most homogeneous, containing 95% uninucleate microspores, 2.5% tetrads and 2.5% bicellular pollen. The BCP population was 77% pure, but also contained some tetrads (3.5%), microspores (12%) and tricellular grains (7.5%). The TCP population comprised 88% tricellular pollen and 12% bicellular pollen. The MPG population was 100% pure with approximately 2% aborted pollen.

### Developmental changes in the male gametophytic transcriptome

*Arabidopsis *ATH1 Genome Arrays were used to explore the dynamics of gene expression throughout male gametophyte development in comparison with sporophytic tissues. Microarrays were hybridized with cRNA probes made from total RNA purified from isolated spores. Hybridization data from two biological replicates derived from independently grown populations of plants were compared. Only genes with a positive hybridization signal and a detection call value of 1 in both experiments were scored as expressed. Microarray data from each pair of replicates were highly correlated, with correlation coefficients of 0.986 (UNM), 0.972 (BCP), 0.991 (TCP) and 0.971 (MPG). Complete microarray data are publicly available at the European *Arabidopsis *Stock Centre (NASC) microarray database [40]. Sporophytic ATH1 Genome Array datasets were downloaded from the NASC website [41]. This provided transcriptome data for seedlings at open cotyledon stage (COT, stage 0.7 [42]), leaves (LEF, stage 6.0), petiole (PET, stage 3.9), stems (STM, stage 6.1), roots (ROT), root hair zone (RHR, stage 1.02), and suspension cell cultures (SUS). Genes that were consistently expressed in replicate sporophytic datasets were identified using the same algorithm used for gametophytic data.

We have previously confirmed and validated the expression pattern of 15 putative pollen-specific genes identified using Affymetrix AG arrays by reverse transcription-PCR analysis [35]. Similarly we validated the current ATH1 datasets by RT-PCR analysis in two separate experiments that included analysis of 41 genes encoding predicted glycosylphosphotidylinositol-anchored proteins (GAPs) [21] and 16 cation/proton exchanger proteins [43]. In both experiments the expression patterns of all genes tested that were identified as pollen-expressed, or pollen-specific by ATH1 analysis were confirmed by RT-PCR.

The ATH1 Genome Array harbors oligonucleotide probes representing 22,591 genes based on the Arabidopsis Genome Initiative annotation. This represents 80.7% of the most recent estimate of 28,000 protein-coding genes in *Arabidopsis *[44]. Of these, 13,977 genes gave a consistently positive expression signal in at least one stage of male gametophyte development, representing 61.9% of the unigene targets on the microarray. The majority of these were expressed in the two earliest developmental stages; 11,565 in microspores and 11,909 in bicellular pollen (Figure 1f). After pollen mitosis II, there was a sharp decline in the number of diverse transcripts to 8,788 in tricellular pollen and 7,235 in mature pollen.

To identify genes expressed preferentially or specifically in developing male gametophytes, hybridization data was compared with sporophytic ATH1 datasets (COT, LEF, PET, STM, ROT and RHR; see Additional data file 1). Transcripts with a consistent positive expression signal in at least one stage of male gametophyte development and a zero signal in any sporophytic dataset were considered male gametophyte-specific. In total, 1,355 specific transcripts were identified, representing 9.7% of the male gametophytic transcriptome. The number of male gametophyte-specific transcripts ranged from 857 (BCP) to 625 (MPG). Thus, in contrast to the decline in the total number of diverse transcripts expressed, the representation of male gametophyte-specific transcripts increased, from 6.9% and 7.2% at UNM and BCP-stages to 8.0% and 8.6% at TCP and MPG-stages respectively.

Analysis of the distribution of transcripts among three abundance classes: high (up to 10-fold less than the maximum signal), medium (10- to 100- fold less) and low (more than 100-fold less) (Figure 1f), revealed a decrease in the proportion of transcripts forming the high-abundance class during development from 20% to 12%. On the contrary, there was sharp increase in the proportion of mRNAs forming the low-abundance class after pollen mitosis II from 4% (UNM) to 14% (MPG). Moreover, 55% of low-abundance transcripts at MPG stage represented repressed mRNAs expressed more abundantly at earlier stages. Thus, the dramatic decrease in the number of transcripts expressed between bicellular and tricellular stages is paralleled by redistribution of mRNA from the high to the low abundance classes. These changes may be associated with reduced cellular activities and cell differentiation processes together with preferential expression of certain classes of genes during pollen maturation. This finding is in accord with the over-representation of cytoskeleton, cell-wall and signaling-related genes that comprise 26% of the high-abundance transcripts at MPG stage. In particular, the average expression signals of cytoskeleton, cell-wall and signaling-related transcripts were increased by 3.1, 3.7 and 2.3-fold, respectively, compared with the UNM stage.

**Figure 2:**
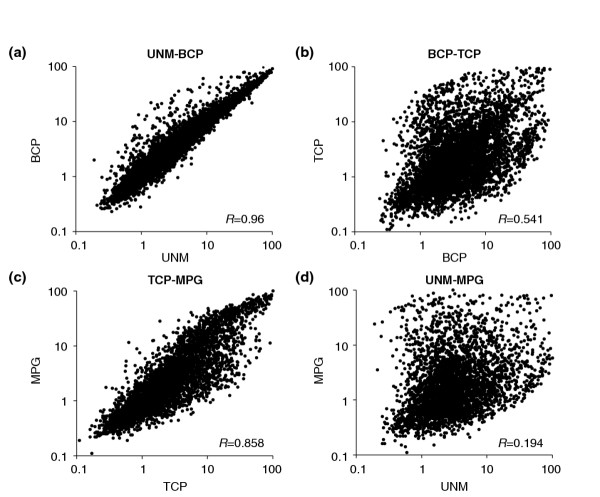
Scatter-plots comparing relative gene expression in pairs of developmental stages. The expression levels of individual genes were normalized using a logarithmic scale of 0 to 100 and genes coexpressed in pairs of transcriptome datasets were plotted. **(a) **UNM versus BCP stage; **(b) **BCP versus TCP stage; **(c) **TCP versus MPG stage; **(d) **UNM versus MPG stage. *R*-value represents the correlation coefficient.

Scatter-plot analysis was used to examine in more detail the complexity of the mRNA decline after PMII. The expression levels of individual genes were normalized using a scale of 0 to 100. Genes coexpressed in pairs of datasets were plotted using a logarithmic scale and a correlation coefficient (*R *value) calculated (Figure 2). There was an extremely high correlation (*R *= 0.96) between the transcriptomes of UNM and BCP, the two earliest developmental stages (Figure 2a). These stages are closely related, with a moderate increase in the expression of a number of genes at BCP stage. The profiles of the two latest developmental stages, TCP and MPG, were also very similar (Figure 2c, *R *= 0.858), but with greater deviation than the early stages. The scatter plot of TCP and MPG revealed the shift between extreme mRNA abundance classes as described above. This was more evident when bicellular and tricellular stages were compared (Figure 2b). The scatter of gene expression values and the low correlation (*R *= 0.541), provide evidence that the major quantitative shift in transcriptome size between BCP and TCP stages is not simply the result of repression, but also involves the activation of new groups of genes associated with pollen maturation. The lack of correlation (*R *= 0.194) between gene expression profiles in uninucleate microspores and mature pollen (Figure 2d), also reflects the pronounced change in cell status from proliferating microspore to terminally differentiated pollen.

The relationship between cell proliferation activities and transcriptome profiles was examined by comparison of early UNM and late MPG stages with a publicly available suspension cell culture dataset. These comparisons demonstrated that the microspore transcriptome was significantly more similar to that of cell suspensions (*R *= 0.474) than to mature pollen (*R *= 0.194). This is also in accord with the lack of correlation between transcriptome profiles of mature pollen and cell suspensions (*R *= 0.13).

### Co-regulated clusters of gametophytic genes

**Figure 3:**
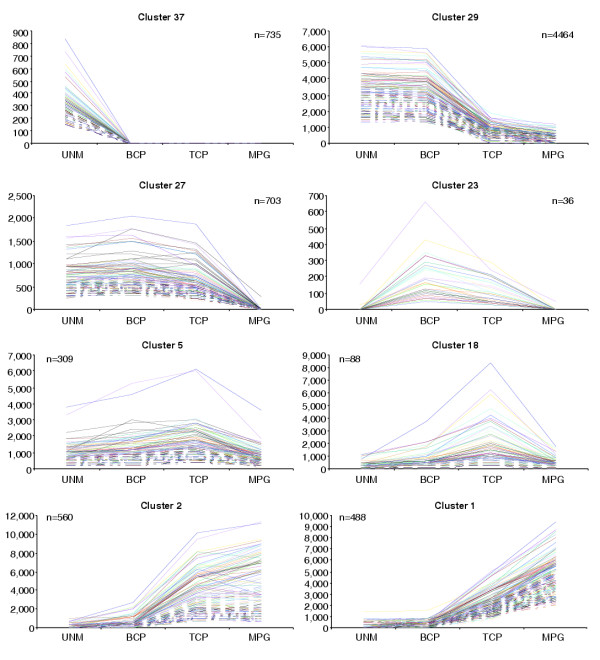
A selection of clusters of genes coexpressed during male gametophyte development. A complete set of all 39 clusters determined using EPCLUST software with a threshold value of 0.05 is available as Additional data files 1 and 2. *n*, number of genes comprising a cluster.

To identify gametophytic genes that may form co-regulated clusters, all 13,977 male gametophyte-expressed genes were hierarchically clustered using EPCLUST clustering and analysis software. Application of a threshold value of 0.05 resulted in the definition of 39 gene clusters covering all phases of male gametophyte development (Figure 3; see also Additional data files 1 and 2). Cluster 37 contained 735 early genes (5.3% of all gametophytic genes) with positive expression signals only at UNM stage. Transcriptome data reflect steady-state mRNA profiles that result from the combination of transcription and mRNA turnover rates. In this regard, some transcripts grouped in early cluster 37 may be inherited through meiosis and/or from the tetrad stage.

The majority of male gametophyte-expressed genes (52%) were grouped into four clusters (25, 27, 29 and 35) comprising early expressed genes repressed after PMII. Several large gene clusters collectively containing 1,899 genes (13.6%) were associated with pollen maturation. These were activated or upregulated between BCP and TCP stages, forming clusters 5, 7, 11, 13, 18-24, 26, 28, 38 and 39. In contrast, a discrete set of 298 genes forming cluster 17 was upregulated only after TCP stage. In total, 3,342 late genes (24%), forming clusters 1-3, 6, 8 and in particular, cluster 17, encode proteins that are likely to function during post-pollination development.

### Expression of regulatory genes throughout male gametophyte development

**Figure 4:**
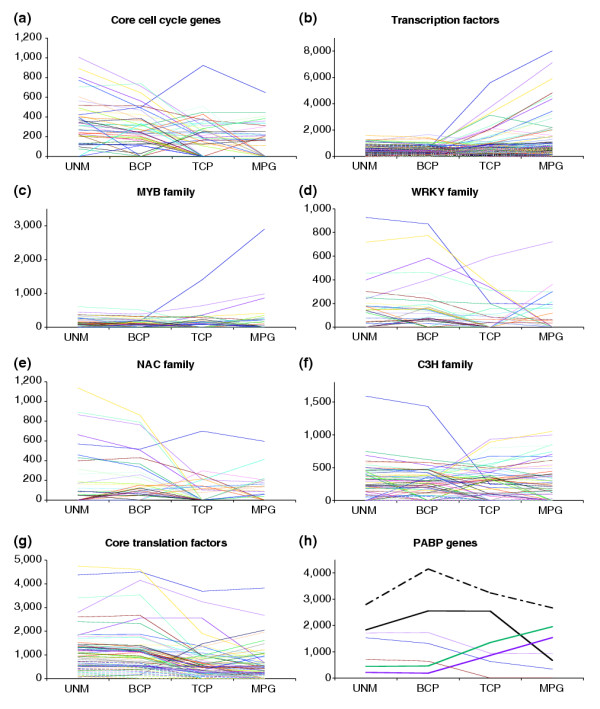
Expression profiles of regulatory genes throughout male gametophyte development. **(a) **Core cell-cycle genes; **(b) **transcription factors; **(c-f) **selected transcription factor families. (c) R2R3-MYB; (d) WRKY; (e) NAC; (f) C3H. Expression profiles of all transcription factor families analyzed are available as Additional data files 1 and 3. **(g) **Core translation factors. **(h) **Poly(A)-binding (PAB) protein genes. Putative male gametophyte-specific (bold) or enhanced (bold dashed) genes are highlighted.

We focused our further analysis on three key categories of genes with likely regulatory significance in male gametophyte development; core cell-cycle genes, transcription factors and core translation factors (Figure 4). Core cell-cycle genes [45] were defined according to TAIR [46]. Genes comprising *Arabidopsis *transcription factor families were derived by compilation of data available at The Ohio State University *Arabidopsis *Gene Regulatory Information Server [47], data published in [48] and databases homology searches. Recent annotations of the MADS-box and bHLH transcription factor gene families were defined according to [49,50], respectively. Core translation factors [51] were defined according to TAIR [46].

#### Core cell-cycle genes

Among 61 core cell-cycle genes, 55 genes were present on the ATH1 GeneChip and 45 (82%) of these were expressed in the male gametophyte (see Additional data file 1). Representative(s) of all families and subfamilies were expressed. The majority of gametophytic core cell-cycle genes showed similar expression profiles (Figure 4a), with a decline in mRNA abundance after UNM stage to zero (or low levels) at TCP and MPG stages. This pattern is consistent with the termination of proliferation of the microspore and generative cell before pollen maturation.

#### Putative transcription factors

We identified 1,594 genes encoding putative transcription factors that were divided into 34 gene families (see Additional data file 1). Their representation on the ATH1 GeneChip was 1,350 (85%). Of these, 608 (45%) were expressed in the male gametophyte, including 54 (15.7%) that were male gametophyte-specific. There were distinct differences in the representation of large transcription factor families (with over 25 members) in the gametophyte. Among those over-represented were the p-coumarate 3-hydroxylase (C3H) family (67% of family members present on the ATH1 GeneChip), the CCAAT family (64%), C2H2 zinc finger proteins (57%), the WRKY family (53%), the bZIP family (51%), the TCP family (50%) and the GRAS family (50%). In contrast, the AUX/IAA (20%), HSF (33%), bHLH (34%), NAC (34%), AP2-EREBP (35%), HB (36%), R2R3-MYB (37%), MADS (37%) and C2C2 zinc finger (37%) gene families were all under-represented.

The dominant expression pattern of transcription factor genes reflected the general repression of mRNA diversity between BCP and TCP stages (Figure 4b). Besides a limited number of constitutively expressed genes, two major transcription factor gene groups could be distinguished. One contained a major group of early-expressed genes and the second a smaller group of genes that were more abundantly expressed late during pollen maturation. The same general tendency was apparent when the profiles of individual transcription factor families were analyzed (exemplified by the C3H family, Figure 4d). Several gene families comprised predominantly early-expressed genes. These were the NAC, WRKY, TCP, ARF, Aux/IAA, HMG-box and Alfin-like gene families (Figure 4c-e, Additional data file 3). Complete lists of transcription factor gene families and their expression profiles are presented in Additional data files 1 and 3.

#### Core translation factors

Among 100 annotated core translation factor genes, 82 were present on the ATH1 GeneChip and 75 (91%) of these were expressed in the male gametophyte (see Additional data file 1). The vast majority of translation factor genes belonged to the early group and these were strongly expressed (Figure 4g). Reflecting the constitutive requirement for protein synthesis, only six genes showed male gametophyte-specific expression. These were: *AtPAB3 *(At1g22760), *AtPAB6 *(At3g16380), *AtPAB7 *(At2g36660), *AteIF2*-*B3 *(At3g07920), *AteIF4G*-like (At4g30680) and *AteIF6*-*2 *(At2g39820). There was a striking over-representation of poly(A)-binding (PAB) proteins among the male gametophyte-specific genes; seven out of eight PAB genes were male gametophyte-expressed, three of which were specific. Moreover, two of these gametophyte-specific PAB genes were among the few late pollen genes encoding translation initiation factors (Figure 4h).

### Integrating transcriptomic and experimental data

**Figure 5:**
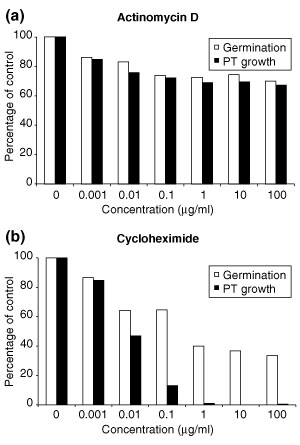
Pollen germination and pollen tube growth *in vitro *in the presence of inhibitors of **(a) **transcription and **(b) **translation. The percentage of germinated pollen and the percentage of pollen capable of extended tube growth were scored independently for each treatment. PT, pollen tube.

The rapid decline of mRNAs encoding translation initiation factors after bicellular stage and the parallel *de novo *synthesis of a new set of late pollen transcription factors, suggested storage of translation factors and ongoing transcription after pollen germination. Therefore we investigated the dependence of *Arabidopsis *pollen germination and tube growth on transcription and translation. Pollen was cultured with increasing concentrations of actinomycin D and cyclohexmide to examine the importance of transcription and translation, respectively. Actinomycin D had only moderate effects on both pollen germination and tube growth even at high concentrations (Figure 5a). Similar results were observed when another transcription inhibitor, cordycepin, was used (data not shown). In contrast, cycloheximide had a dramatic effect on pollen tube growth (Figure 5b). The presence of 0.1 μg/ml cycloheximide only inhibited pollen germination by 40%, but pollen tube growth was inhibited by 90%. At higher concentrations, 40% of pollen was still able to germinate, but further pollen tube growth was blocked. We conclude that active pollen tube growth is strictly dependent upon protein synthesis, and that pollen germination and tube growth are relatively independent of transcription.

## Discussion

To identify patterns of gene expression involved in *Arabidopsis *male gametophyte development, we compared the transcriptomes of isolated spores at four discrete developmental stages using ATH1 microarrays. ATH1 microarrays harbor probe sets for 22,591 annotated genes [52]. Of these, 61.9% (13,977 genes) gave positive hybridization signals in at least one stage of male gametophyte development. A comparable proportion of active genes was reported for isolated root cells which expressed 10,492 genes (46%) on ATH1 microarrays [8]. Moreover, in similar studies of animal cell development, 53% of 13,179 arrayed genes were found to be expressed during early murine adipocyte differentiation [1].

As the proportion of known genes embedded on the ATH1 array is 80.7%, we estimate the total number of genes expressed throughout *Arabidopsis *male gametophyte development to be more than 17,000. Similarly, the total number of genes expressed at individual developmental stages is estimated to be 14,300 at UNM stage, 14,800 at BCP stage, 10,900 at TCP stage and 9,000 at MPG stage. Previous gene-by-gene approaches identified only 21 different genes expressed during *Arabidopsis *male gametophyte development (for a review see [16]). Moreover, only three of these genes were shown to be expressed at microspore stage [53-55]. The data sets reported here include more than 11,000 microspore-expressed genes, representing a 3,600-fold increase in knowledge of gene expression in haploid microspores.

Two recent studies of the *Arabidopsis *mature pollen transcriptome using Affymetrix 8K AG arrays led to the identification of 992 and 1,584 pollen-expressed mRNAs, respectively [35,36]. Results obtained with ATH1 and AG arrays are considered comparable and largely independent of the different probe sets used [56]. However, there was a significant discrepancy in the number of incorrectly annotated genes between both arrays, with 6.3% of probe sets on the AG array being incorrectly annotated in comparison with only 0.4% on the ATH1 array [56]. Therefore, results from ATH1 arrays are more accurate as well as more comprehensive. Accordingly, the use of the more complete ATH1 array and more accurate microarray normalization protocols led to an increase in the estimated total number of genes expressed in mature pollen from around 3,500 [35] to around 9,000 (this study). The proportion of these genes that are considered male-gametophyte specific is strongly dependent on the choice of the set of reference sporophytic datasets. In the work reported here, the availability of more comprehensive sporophytic datasets and the application of more stringent criteria therefore led to a decrease in the estimated number of putative pollen-specific genes from around 1,400 [35] to around 800 (this study). This number could be reduced further if cell-type-specific expression within an organ limits detection of overlap with pollen expression. Our data highlight the extensive overlap between sporophytic and gametophyte gene expression and reveal the subset of the transcriptome that is strongly enhanced or specifically expressed during male gametophyte development. Considering all stages of microsporogenesis the total number of putative male-gametophyte-specific genes was 1,355 with the proportion of specific genes increasing from 6.9% at UNM-stage to 8.6% at MPG-stage. Among the male-gametophyte-specific genes identified there was an increase in the collective proportion of cell-wall, cytoskeleton, signaling and transport-related genes from 22% at UNM stage to 34% in MPG stage. This reflects the increasing functional specialization of mature pollen in preparation for a dramatic change in the pattern of cell growth during pollen germination and pollen tube growth.

Developmental analysis of transcriptome data revealed two striking features, a sharp reduction in transcript diversity after BCP stage and a major shift in mRNA populations between BCP and TCP stages. The decline in mRNA diversity after BCP stage is associated with terminal differentiation as well as the documented phenomenon of protein storage in pollen (see [57], and this study). Moreover, this large-scale repression associated with termination of cell proliferation after PMII is accompanied by the selective activation of new groups of genes that are likely to function during pollen maturation and post-pollination development.

It is interesting that the expression profiles of UNM stage and BCP stages are similar despite the presence of two different cell types in pollen grains at BCP stage - the larger vegetative cell and the smaller generative cell. Given the limited volume of cytoplasm associated with the generative cell, developmental changes in gene expression in the gametic or male germline cells are likely to be masked by the predominant contribution of the vegetative cell cytoplasm. Therefore, our male gametophytic gene expression profiles largely reflect the passage of the microspore through cell division and changes in gene expression associated with the differentiation of the vegetative cell. Large-scale changes in gene expression occur between BCP and TCP stages, and therefore do not coincide with asymmetric division of the microspore. UNM expression patterns persist into the bicellular stage, which is consistent with experiments that demonstrate that vegetative cell fate is specified independently of cell division at pollen mitosis I [58].

In contrast, generative cell fate appears to be dependent on asymmetric division at pollen mitosis I [25,58]. Sperm-cell cDNAs and databanks recently established in maize, lily, tobacco and *Plumbago zelanica *[30-33] provide valuable gametic gene-expression data in other species. Although our data do not provide direct information about gametic gene expression in *Arabidopsis*, further development of cell gamete isolation sorting [36] would allow genome-wide identification of generative- and sperm-cell-specific genes in comparison with the datasets generated here.

Hierarchical cluster analysis provided detailed evidence for the dramatic switch between early and late developmental programs. We identified 39 gene clusters that could correspond to co-regulated genes. These included early clusters, several clusters of late genes, those with constitutive expression profiles and clusters showing transient expression with peaks at BCP or TCP stages. The large size of cluster 29 (4,464 genes) documents the homogeneity in expression profiles of most early genes. In contrast, late gene clusters included a significant number of genes with similar profiles between BCP and TCP stages, followed by expression profiles that deviated between TCP and MPG stages. Cluster 1, and in particular cluster 17, contained genes strongly upregulated in TCP and MPG, with likely functions in post-pollination events. The differential fate of certain late gene clusters is likely to be a feature of their requirement during pollen maturation or post-pollination events.

Our analysis revealed completely different expression profiles of transcription factors when compared to core translation factors. The majority of core translation factors belonged to the early-group genes with few that were male gametophyte-specific. This may be expected, given that many genes are involved in general cellular activities. However, genes encoding PAB proteins did not follow the general trend. Seven out of eight *Arabidopsis *PAB mRNAs were gametophytically expressed. Three PAB genes (*PAB3*, *PAB6 *and *PAB7*) appeared to be male gametophyte-specific and *PAB5 *was preferentially expressed in pollen. Moreover, *PAB3 *and *PAB5 *are the most abundant early and constitutive PAB mRNAs and *PAB6 *and *PAB7 *belong among the few late core translation-factor genes. Although these data suggest-specific expression, our data do not rule out expression in other sporophytic tissues, particularly in flowers. Indeed, previously published expression data confirmed the expression of these PABs in other reproductive tissues together with pollen [59].

Conversely, transcription factors showed more diverse spectra of expression profiles including early, constitutive and late. There was a considerable variation in the expression profiles of individual transcription factor families. The most over-represented was the C3H family, members of which are known to have roles in lignin and other phenylpropanoid pathways in plants [60]. Although sporopollenin synthesis is believed to be under strict sporophytic control (see [16]), the diversity of gametophytic C3H transcription factors might suggest a function for these genes in regulating chemical interactions between phenylpropanoid precursors secreted by the tapetum. One candidate is the At1g74990 gene encoding a putative RING finger protein, which is abundantly and preferentially expressed at UNM and BCP stages.

The majority of core translation factors belonged to the early gene clusters. In contrast, a significant number of transcription-factor genes were strongly expressed during pollen maturation. These data alone did not obviously support the fact that pollen germination and early tube growth in many species are largely independent of transcription, but vitally dependent on translation [61]. Similarly, we found that *Arabidopsis *pollen germination and tube growth were relatively independent of transcription, and that active pollen-tube growth, and to a lesser extent pollen germination, were dependent upon protein synthesis. It is known for some plant species that mRNAs and rRNAs accumulate during pollen maturation and are stored for use during pollen germination [62,63]. Our results show that *Arabidopsis *pollen is charged with a diverse complement of stored mRNAs that could be used to support pollen germination and pollen tube growth. Moreover, the early synthesis of mRNAs encoding translation factors strongly suggests that these are preformed and stored in mature pollen grains to support rapid activation upon hydration and germination. We also suggest that some abundant late transcription factors could regulate maturation-associated genes or act as repressors of inappropriate transcription in growing pollen tubes.

## Conclusions

The key impact of this work is that it provides a genome-wide view of the complexity of gene expression during single cell development in plants. Analysis of the male gametophytic transcriptome provides comprehensive and unequivocal evidence for the unique state of differentiation that distinguishes the developing male gametophyte from the sporophyte. Male gametogenesis is accompanied by large-scale repression of gene expression that is associated with the termination of cell proliferation and the selective activation of new groups of genes involved in maturation and post-pollination events. Development is associated with major early and late transcriptional programs and the expression of about 600 putative transcription factors that are potential regulators of these developmental programs. This wealth of information lays the foundation for new genomic-led studies of cellular functions and the identification of regulatory networks that operate to specify male gametophyte development and functions.

## Materials and methods

### Plant material and spore isolation

*Arabidopsis thaliana *ecotype Landsberg *erecta *plants were grown in controlled-environment cabinets at 21°C under illumination of 150 μmol/m^2^/sec with a 16-h photoperiod. Isolated spores from three stages of immature male gametophyte were obtained by modification of the protocol of Kyo and Harada [38,39]. After removal of open flowers, inflorescences from 400 plants were collected and gently ground using a mortar and pestle in 0.3 M mannitol. The slurry was filtered through 100 μM and 53 μM nylon mesh. Mixed spores were concentrated by centrifugation (50 ml Falcon tubes, 450 *g*, 3 min, 4°C). Concentrated spores were loaded onto the top of 25%/45%/80% Percoll step gradient in a 10-ml centrifuge tube and centrifuged (450 *g*, 5 min, 4°C). Three fractions were obtained containing: (1) microspores mixed with tetrads; (2) microspores mixed with bicellular pollen; and (3) tricellular pollen (Figure 1). Fraction 2 was diluted with one volume of 0.3 M mannitol loaded onto the top of a 25%/30%/45% Percoll step gradient and centrifuged again under the same conditions. Three subfractions of immature pollen were obtained: (2.1) microspores; (2.2) microspores and bicellular pollen mixture; and (2.3) bicellular pollen. Spores in each fraction were concentrated by centrifugation (eppendorf tubes, 2,000 *g*, 1 min, 4°C) and stored at -80°C. The purity of isolated fractions was determined by light microscopy and 4',6-diaminophenylindole (DAPI) staining according to [25]. Viability was assessed by fluorescein 3',6'-diacetate (FDA) treatment [58]. Mature pollen was isolated as described previously [35]. Pollen tubes were cultivated *in vitro *for 4 h according to [21]. Pollen was scored as germinated when pollen tubes were longer than half a pollen grain diameter. Pollen-tube growth was scored by counting those with tubes longer than two pollen grain diameters.

### RNA extraction, probe preparation and DNA chip hybridization

Total RNA was extracted from 50 mg of isolated spores at each developmental stage using the RNeasy Plant Kit (Qiagen) according to the manufacturer's instructions. The yield and RNA purity was determined spectrophotometrically and using an Agilent 2100 Bioanalyzer at the NASC.

Biotinylated target RNA was prepared from 20 μg of total RNA as described in the Affymetrix GeneChip expression analysis technical manual. Double-stranded cDNA was synthesized using SuperScript Choice System (Life Technologies) with oligo(dT)_24 _primer fused to T7 RNA polymerase promoter. Biotin-labeled target cRNA was prepared by cDNA *in vitro *transcription using the BioArray High-Yield RNA Transcript Labeling Kit (Enzo Biochem) in the presence of biotinylated UTP and CTP.

*Arabidopsis *ATH1 Genome Arrays were hybridized with 15 μg labeled target cRNA for 16 h at 45°C. Microarrays were stained with streptavidin-phycoerythrin solution and scanned with an Agilent 2500A GeneArray Scanner.

### Data analysis

Sporophytic data from public baseline GeneChip experiments used for comparison with the pollen transcriptome were downloaded from the NASC website [41,64]. The list of dataset codes was as follows: COT (three replicates), Cornah_A4-cornah-wsx_SLD_REP1-3; LEF (three replicates), A4-LLOYD-CON_REP1-3; PET (three replicates), Millenaar_A1-MILL-AIR-REP1-3; STM (two replicates), Turner_A-7-Turne-WT-Base1-2_SLD; ROT (two replicates), Sophie_A1-Fille-WT-nodex_SLD, Sophie_A5-Fille-WT-nodex_SLD; RHR (two replicates), Jones_A1-jones-WT1, SLD, Jones_A1-jones-WT2_SLD; SUS (three replicates), A1-WILLA-CON-REP1-3.

All gametophytic and sporophytic datasets were normalized using freely available dChip 1.3 software [65]. The reliability and reproducibility of analyses was ensured by the use of duplicates or triplicates in each experiment, the normalization of all 26 arrays to the median probe intensity level and the use of normalized CEL intensities of all arrays for the calculation of model-based gene-expression values based on the Perfect Match-only model [66,67]. A given gene was scored as 'expressed' when it gave a reliable expression signal in all replicates. Expression signal value '0' means that the detection call value was not 'present' in all replicates provided. All raw and dChip-normalized gametophytic datasets are available at the Institute of Experimental Botany AS CR website [68]. Although a RT-PCR validation of microarray data was not performed specifically for the purpose of this publication, our confidence in the quality of the data presented is based on our previously published RT-PCR validation of the expression of 70 genes [21,35,41].

Microsoft Excel was used to manage and filter the microarray data. For annotation of genes present on the ATH1 Array, the *Arabidopsis *Genome Annotation Release 3.0 published by The Institute for Genomic Research [52] was used. Genes were sorted into functional categories created according to data mined from the Munich Information Center for Protein Sequences *Arabidopsis thaliana *Database [69], Kyoto Encyclopedia of Genes and Genomes [70] and TAIR [46]. Hierarchical clustering of expressed genes was performed using expression-profile data clustering and analysis software EPCLUST [71], with correlation measure based distance and average linkage clustering methods.

## Additional data files

The following additional data is available with the online version of this article: Additional data file 1 is an Excel file containing the following items. The table Data contains the complete transcriptomic datasets used. Data were normalized using dChip 1.3 as described in Materials and methods. Expression signal value '0' means that the detection call value for particular gene was not 'present' in all replicates provided. In the column 'Cluster', the appropriate cluster for each male gametophyte-expressed gene is shown. The table Clusters gives the number of genes comprising all 37 clusters of genes coexpressed during male gametophyte development. The table Cell-cycle data lists core cell-cycle genes showing their expression values in male gametophytic datasets. Genes were defined according to [21]. The chart shows expression profiles of male gametophyte-expressed core cell-cycle genes. The table Transcription data lists transcription-factor genes, showing their expression values in male gametophytic datasets. Genes comprising *Arabidopsis *transcription factor families were derived by compilation of data available at the Ohio State University Arabidopsis Gene Regulatory Information Server [47], data published in [22] and database homology searches. MADS-box and bHLH gene families were defined according to [23] and [24], respectively. The table Translation data lists core translation-factor genes showing their expression values in male gametophytic datasets. Genes were defined according to the FIAT database [51]. The chart shows expression profiles of male gametophyte-expressed core translation-factor genes. The Transcription table summarizes transcription factor gene families showing the number of genes expressed during male gametophyte development. Additional data file 2 lists a complete set of 39 clusters of genes coexpressed during male gametophyte development. Clusters were determined using EPCLUST software with a threshold value of 0.05. The list of genes comprising each cluster is given in Additional data file 1. Additional data file 3 gives the expression profiles of male gametophyte-expressed transcription factors sorted into individual gene families. Expression data are given in Additional data file 1.

## Supplementary Material

Additional data file 1 The complete transcriptomic datasets used Click here for additional data file

Additional data file 2 The complete set of 39 clusters of genes coexpressed during male gametophyte development Click here for additional data file

Additional data file 3 The expression profiles of male gametophyte-expressed transcription factors sorted into individual gene families Click here for additional data file
