# Supplementary figures and images for: Transcriptome analysis of haploid male gametophyte development in Arabidopsis
Source: Genome Biol. 2004 Oct 27;5(11):R85. doi: 10.1186/gb-2004-5-11-r85 (PMC545776; doi:10.1186/gb-2004-5-11-r85)

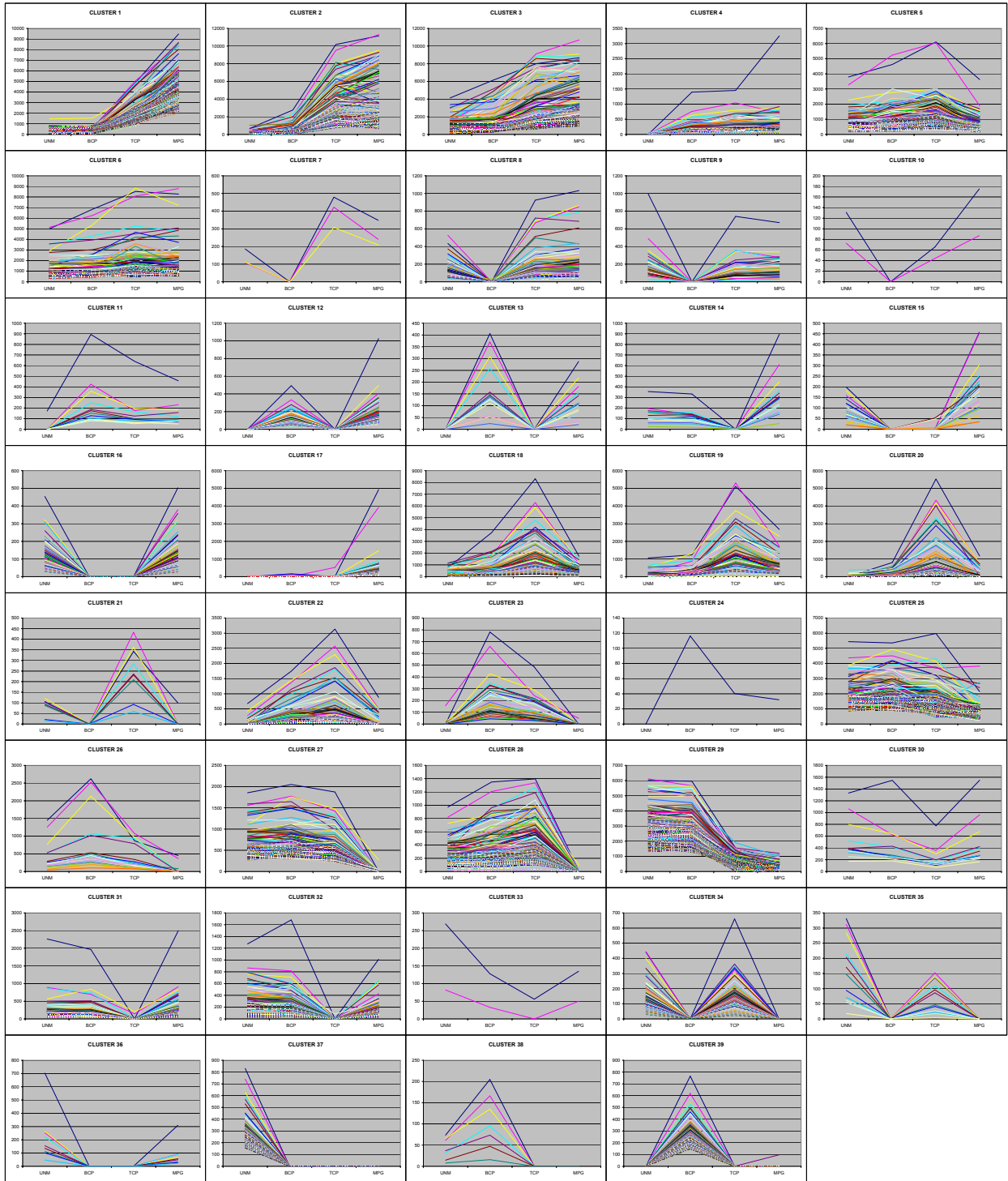

Supplement: Additional data file 2 — The complete set of 39 clusters of genes coexpressed during male gametophyte development [file gb-2004-5-11-r85-s2.pdf]

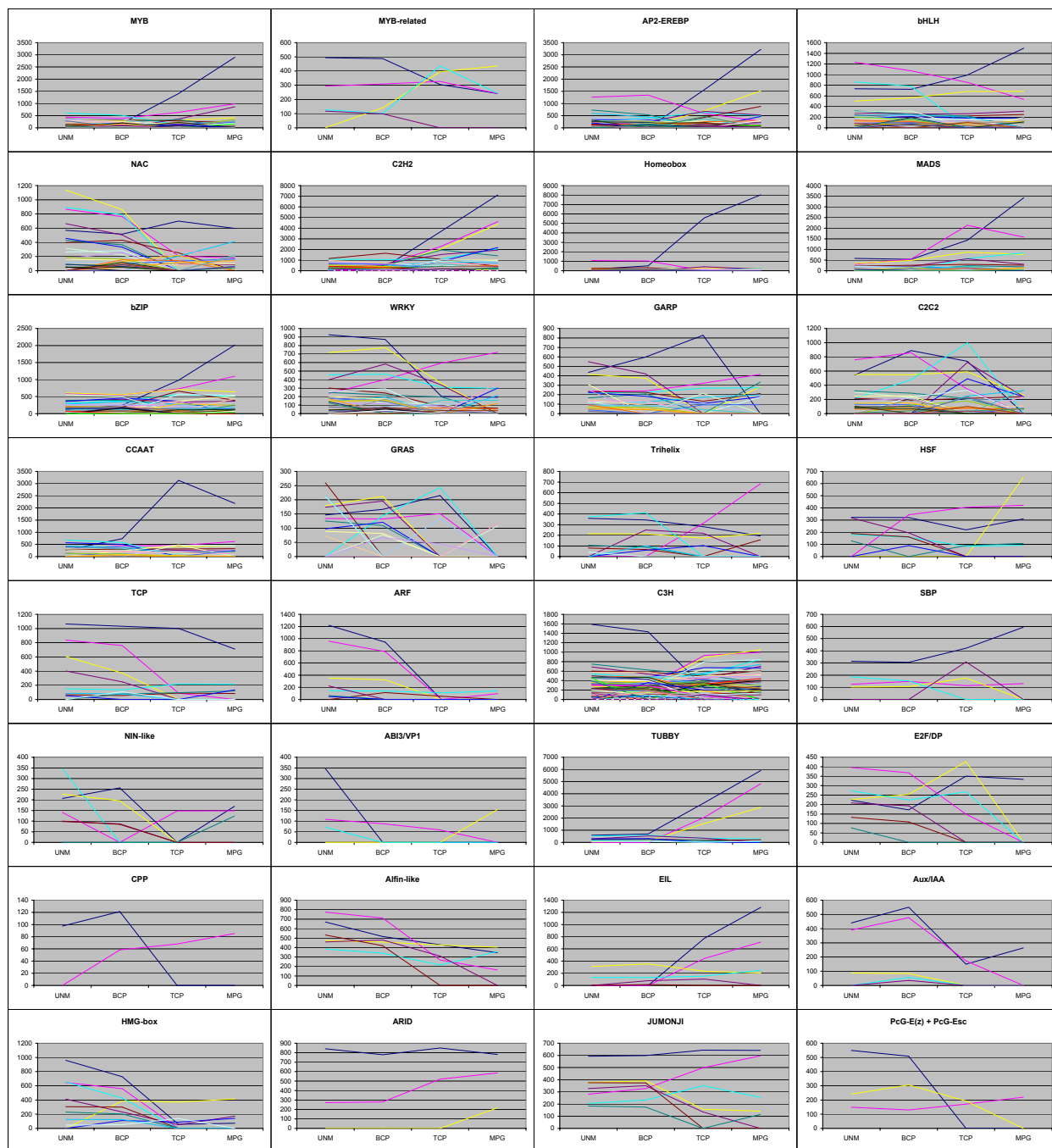

Supplement: Additional data file 3 — The expression profiles of male gametophyte-expressed transcription factors sorted into individual gene families [file gb-2004-5-11-r85-s3.pdf]
